# Assessment and verification of commercially available pressure cookers for laboratory sterilization

**DOI:** 10.1371/journal.pone.0208769

**Published:** 2018-12-11

**Authors:** Vaille A. Swenson, Amanda D. Stacy, Michael O. Gaylor, Blake Ushijima, Benjamin Philmus, Loralyn M. Cozy, Nina M. Videau, Patrick Videau

**Affiliations:** 1 Biology Department, Dakota State University, Madison, South Dakota, United States of America; 2 Department of Biology, Southern Oregon University, Oregon, Ashland, United States of America; 3 Chemistry Department, Dakota State University, Madison, South Dakota, United States of America; 4 Carlson College of Veterinary Medicine, Oregon State University, Corvallis, Oregon, United States of America; 5 Department of Pharmaceutical Sciences, College of Pharmacy, Oregon State University, Corvallis, Oregon, United States of America; 6 Department of Biology, Illinois Wesleyan University, Bloomington, Illinois, United States of America; 7 Bowman Gray Center for Medical Education, Wake Forest University, Winston-Salem, North Carolina, United States of America; Fujian Agriculture and Forestry University, CHINA

## Abstract

Laboratory science requires careful maintenance of sterile reagents and tools as well as the sterilization of waste prior to disposal. However, steam autoclaves typically used for this purpose may not be readily accessible to everyone in the scientific community, such as K-12 teachers, researchers in the field, students in under-funded laboratories, or persons in the developing world who lack funding and resources. This work examines the use of commercial electric pressure cookers as an alternative method for the sterilization of media, instruments, and waste. Four commonly available brands of pressure cooker were tested for their ability to sterilize microbiological media, a variety of metal instruments, and high-titer microbial cultures. All four pressure cookers were able to sterilize these starting materials as well as a range of microbial types, including Gram-positive bacteria, Gram-negative bacteria, filamentous fungi, unicellular fungi, and mixed environmental samples. Only the Instant Pot, however, was able to sterilize autoclave tester ampoules of *Geobacillus stearothermophilus* spores. These results suggest that, depending on the nature of the work undertaken, store-bought pressure cookers can be an appropriate substitute for commercial autoclaves. Their adoption may also help increase the accessibility of science to a broader range of investigators.

## Introduction

Laboratory research, medical treatment, industrial production of pharmaceuticals, and a wide variety of other scientific applications all rely on sterilization procedures to protect workers and consumers from pathogenic contaminants. This may manifest as the maintenance of axenic strains for study and storage, the preparation of surgical devices, the growth of large cultures for compound extraction and purification, among others. Even space exploration labs work to sterilize equipment that will land on other planets to prohibit the introduction of Earth microbes capable of colonization and, possibly, destruction. A strict definition of sterilization requires every organism to be inactivated following a sterilization procedure [1]. A more realistic definition of sterility stipulates that, if sterile, no growth will be observed upon incubation in appropriate growth medium. This second definition is likely more accurate when considering the level of sterility achieved in most laboratory or medical situations because it may not be possible to verify that every contaminant is inactivated, but observing outgrowth, or lack thereof, is usually possible. To this end, the World Health Organization, U.S. Pharmacopeia, International Organization for Standardization, U.S. Food and Drug Administration, and others have set forth standards that must be met to consider a surface or item sterile for human use.

Various methods are available to sterilize equipment and surfaces, including ethylene oxide gas, compounds like phenol, glutaraldehyde, peracetic acid, and hydrogen peroxide, dry heat, UV irradiation, and steam sterilization [2]. Steam sterilization is usually conducted in an autoclave that reaches and maintains 121°C and 15 PSI for a desired duration, often at least 30 min. In other cases, however, longer sterilization times at temperatures as low as 115°C are considered appropriate to inactivate contaminants. The use of autoclaves as sterilization devices is ubiquitous across laboratories and medical facilities because of the short time required for sterilization, the ease of training and use of the equipment, the absence of harmful chemicals, and the consistent reproducibility as a means of pathogen control [3]. To verify that an autoclave is consistently reaching the desired temperature and pressure for sterilization, biological tests are regularly conducted to demonstrate that the most heat-resistant organisms are inactivated, which implies that all other more sensitive contaminants would be similarly sterilized [4]. In one of the more common tests, a suspension of especially heat-resistant *Geobacillus stearothermophilus* (originally deposited as *Bacillus stearothermophilus* Donk) endospores is autoclaved and growth is assessed either by plate-based growth or by the appearance of turbidity and color change of an indicator dye [5, 6]. Failure of the endospores to germinate and grow indicates that the autoclave is functioning properly.

Failure to achieve sterile materials, especially those that are to be re-used (such as surgical instruments, tools like bronchoscopes, and autografts in medicine and culture glassware in laboratories) may result from many user- and machine-oriented causes. A serious example of user error in sterilization was described by Dancer et al; an outbreak of surgical site infections in orthopaedic patients and endophthalmitis in ophthalmology patients occurred in sequence despite having relatively little overlap in specialty surgical instruments used by the two departments [7]. Much more common causes of equipment sterilization failure are due to flaws in the machine itself, as evidenced by reports of primary inoculation tuberculosis after contaminated acupuncture needles were used, a *Pseudomonas aeruginosa* outbreak after steam sterilization inadequately removed residual material from arthroscopic tools, a *Mycobacterium chelonae* outbreak after laparoscopic instruments were chemically disinfected but subsequently rinsed in trays containing a *M*. *chelonae* biofilm, and a similar outbreak of *M*. *chelonae* in a private plastic surgery clinic after liposuction tubing was colonized with a biofilm [8–11]. Though these published accounts highlight sterilization mishaps in medicine, equally common are those that occur in the lab, however, they are rarely published because the consequences are relatively minor. These incidents of contamination leading to pathology illustrate the need for sterilization and verification of the means of achieving it.

While steam sterilization is a common means of decontaminating instruments and surfaces, autoclaves can be prohibitively expensive for underfunded research and teaching facilities. Additionally, the cumbersome size and weight of both floor and benchtop autoclaves hinders their transportation to field sites. To provide a possible sterilization alternative to large pricey autoclaves, we assessed the viability of using electric, self-contained pressure cookers to sterilize laboratory instruments and media and inactivate high titers of various microbes. Of the four brands of 8-quart pressure cookers tested, all were able to inactivate contaminants in media, consumables, and fungal and bacterial cultures within a maximum of 60 mins of run time. In this work, run time is the time spent at the operating temperature and pressure of the pressure cooker likely resulting in sterilization. Only the Instant Pot brand pressure cooker was able to inactivate *G*. *stearothermophilus* endospores, which indicated that it would be the most appropriate choice for a laboratory pressure cooker. These results suggest that pressure cookers can sterilize laboratory components sufficiently for use in a relatively short timeframe, which may make sterilization available to many more groups worldwide.

## Materials and methods

### Bacterial growth conditions

All bacterial and fungal strains used in this study are listed in Table 1. Lysogeny Broth-Miller (LB), Trypticase Soy Broth (TSB), Nutrient Broth (NB) for bacterial growth, and Sadouraud Dextrose Broth (SDB) for fungal growth were purchased from Becton Dickenson and prepared according to the manufacturer’s instructions. Yeast extract-peptone-dextrose (YPD) medium for fungal growth was prepared as previously described [12]. All media were solidified with the addition of 1.5% (w/v) agar, and strains were grown in the appropriate medium at the temperature and for the duration specified in Table 1. Strains were stored at -80°C in 20% glycerol stocks, which were streaked on a plate to visually assess purity prior to inoculation for growth in large cultures. Liquid cultures were aerated with shaking at 150 rpm. All media and glassware were sterilized in an autoclave prior to use, and manipulations were conducted in a laminar flow hood to maintain sterility.

**Table 1:** All strains used in this study, their growth conditions and characteristics, and the time required to sterilize them in all pressure cookers tested. The CFU/mL presented for each strain was determined following the 24–72 h of growth time allotted for each strain.

Organism	Source	Media	Growth time in culture (hours)	Growth temperature (°C)	CFU/mL	Minimum sterilization time (mins)
*Bacillus subtilis* subsp. *subtilis*	ATCC 6051	NB	24	30	1.5 x 10^7^	15
*Candida albicans* 3147	ATCC 10231	YPD	48	30	7 x 10^7^	60
*Escherichia coli* K-12	Carolina 155068	NB	24	37	5.3 x 10^7^	15
*Mycobacterium smegmatis* mc(2)155	ATCC 700084	TSB	72	37	1.1 x 10^8^	15
*Pantoea agglomerans* Eh 355	Gift [13]	LB	48	27	2.6 x 10^8^	15
*Penicillium sp*.	Lab isolate	SDB	72	30	1.2 x 10^5^	60
*Pseudomonas aeruginosa*	Carolina 155250A	LB	24	37	9.3 x 10^7^	15
*Saccharomyces cerevisiae* VL6-48	ATCC MYA-3666	YPD	48	30	7.7 x 10^7^	60
*Staphylococcus aureus*	Carolina 155554A	TSB	24	37	7.6 x 10^8^	15
*Vibrio cholerae* O1 biovar El Tor N16961	ATCC 39315	LB	24	37	1.8 x 10^10^	15

Abbreviations: LB, lysogeny broth-Miller; NB, nutrient broth; SDB, Sabouraud dextrose broth; TSB, trypticase soy broth; YPD, yeast-extract potato dextrose broth.

### Pressure cooker sterilization

The 8-quart pressure cookers used in this study were operated according to the manufacturer’s instructions (Table 2). The manual slow cooking setting at high temperature and pressure was used for sterilization, and 0.5 L of deionized water was added to each run to generate steam. The pressure cookers were vented within five mins of the completion of each run to quickly depressurize the instrument rather than allowed it to slowly exhaust and maintain a prolonged sub-sterilization temperature. At maximum capacity, 1.5 L of liquid could be sterilized during each run in the following configuration: a 1.5 L beaker filled to 1 L and two 0.5 L beakers filled to 0.25 L each with all beakers covered with aluminum foil. This arrangement facilitated sterilization of the maximum amount of liquid per run while preventing overflow from the beakers. For every condition, sterilization trials were conducted in triplicate in each pressure cooker.

**Table 2:** Pressure cookers utilized in this study. Prices are as advertised on Amazon.com and working pressures and temperatures are found in their respective user manuals.

Pressure cooker brand	Model number	Working Pressure (PSI)	Working Temperature (°C)	Price paid in January, 2018	Price in June, 2018
COSORI	CP018-PC	5.8–10.0	113–115	$120.93	$89.99
Gourmia	GPC-800	Up to 10.2	N/D*	$129.99	$79.99
GoWISE	22623	7.2–13.0	88–99	$80.10	$79.96
Instant Pot	IP-DUO80	10.2–11.6	115–118	$129.99	$139.99

*Not disclosed in the operating manual.

To sterilize media for culture, 1.5 L of liquid media was prepared and portioned out to the three beakers, and the appropriate amount of agar and a stir bar were added to make solid media. Liquid media were allowed to cool on the bench while solid media were cooled on a stir plate prior to pouring into Petri dishes in a laminar flow hood. A positive control batch of media was sterilized in the autoclave and a negative control batch was prepared and not sterilized. Following treatment, all media were incubated at 30°C for one week and microbial growth was assessed visually such that optically transparent, non-turbid liquid media and plates lacking observable colonies were considered the result of proper sterilization.

To inactivate microbes, liquid cultures of each strain in Table 1 were grown such that 1.5 L of liquid could be sterilized in each pressure cooker in triplicate. Following growth for the required time and temperature to attain a dense culture, a 10-fold serial dilution series from 10^0^−10^−9^ was conducted in triplicate and plated on the same media used for liquid culture to determine the CFU/mL. After each trial, 1 mL aliquots from each culture were plated on the same media used for liquid culture in triplicate, incubated at the culture temperature for one week (Table 1), and sterilization was determined visually by the lack of detectable colonies. To inactivate an uncharacterized heterogeneous mixture of microbes, soil was collected from the Dakota State University campus (Lat, Long: 44.01288, -97.1115) and resuspended at 20 mg/L in sterile MilliQ water. After 20 mins of stirring to create a slurry, the mixture was allowed to settle for 30 mins, and the liquid was decanted into a sterile beaker for continued use (hereafter referred to as soil water) while the particulate was left behind. The CFU/mL was determined by preparing an identical dilution series to that created above, and plating on both nutrient agar (NA) and Sabouraud dextrose agar (SDA) to account for the presence of bacteria and fungi in the mixture. The post-sterilization liquid was also plated and grown at 30°C as above on both NA and SDA. A positive control, soil water sterilized in the autoclave, and a negative control, non-sterilized soil water, were prepared for direct comparison to the sterilization experiments performed in the pressure cookers.

A 15-cm Mall probe and seeker, 15-cm reagent digger spatula, and dissection scissors, all steel, were chosen as equipment for sterilization. Each metal implement was dipped in soil water to coat it in microbes and then prepared in two ways: 1) wrapped in at least two layers of aluminum foil, and stood upright in a beaker for sterilization, and 2) placed into sterile 25 mm x 150 mm culture tubes and stood upright in a culture tube racks for sterilization. Once the run was complete, the Mall probe and seekers and reagent digger spatulas sterilized in foil were introduced into sterile 25 mm x 150 mm culture tubes, while the scissors were placed in sterile 0.3 L beakers. The instruments were submerged in 30 mL or 120 mL of either NB or SDB in culture tubes or 0.3 L beakers, respectively, in triplicate for each media type. Submerged metal instruments were incubated at 30°C for a week and sterilization was determined visually by the presence of optically transparent, non-turbid liquid media; contaminated instruments resulted in turbid microbial growth in both NB and SDB. These metal instruments were also sterilized in the autoclave and negative control instruments were prepared and not sterilized.

Spore ampoules containing 1 mL of 10^6^ *Geobacillus stearothermophilus* spores (CA# SA1-15-06) and 1 mL negative control ampoules (CA# SA1-NC-10) were used according to the manufacturer’s instructions (Crosstex). A single spore ampoule was tied with string to a metal washer to weight it to the bottom of 1 L of deionized water in a 1.5 L beaker, along with two 0.5 L beakers each holding 0.25 L of deionized water, in each pressure cooker. After sterilization, the water was decanted and the ampoules were incubated upright at 60°C for 48 h. Sterilization was indicated by a purple, non-turbid liquid within the ampoule while non-sterilized ampoules turned yellow and became turbid with growth within 24 h of incubation at 60°C. Spore ampoules were also sterilized in the autoclave, a negative control spore ampoule was not sterilized and incubated to demonstrate the result of *G*. *stearothermophilus* growth, and a negative control ampoule lacking spores was included for comparison.

### Isolation and identification of a fungal isolate

Following several weeks of growth on a BG-11 + 5% (w/v) sucrose plate harboring *Anabaena* sp. strain PCC 7120, a fungal colony was observed that inhibited growth of the cyanobacterium [14]. This fungus was streaked on BG-11 + 5% (w/v) sucrose plates for isolation several times until a uniform morphology was observed. Fungal genomic DNA was extracted by phenol chloroform extraction as previously described [15], and the internal transcribed spaces (ITS) 1 and 2 and the 5.8S rRNA within the ribosomal operon were amplified from this genomic DNA by PCR with the primers ITS1 and ITS4 [16]. The resulting 572 bp fragment was sequenced via Sanger sequencing at the Center for Genome Research and Biocomputing at Oregon State University and was deposited into GenBank under accession number MH734615. Based on similarity to isolates of *Penicillium citrinum* as determined by BLAST search [17], 28 other sequences were aligned using Muscle [18]. Phylogenetic analysis was performed using the Maximum Likelihood method and the generalized time-reversible (GTR) algorithm with 1000 bootstrap replicates in MEGA7 [19, 20]. Sequence alignment for presentation was conducted using Clustal Omega (1.2.4) with the default parameters [21].

## Results

### Pressure cookers sterilize culture media

Recent years have seen increasing popularity of the use of programmable pressure cookers to plan and expedite home cooking. This likely stems from the pressure cooker’s utility as a multifaceted kitchen appliance, an integrated design that combines a heating element with internal controls to precisely maintain different cooking temperatures and times, and their inexpensive price around $100 USD. The rise in popularity has been accompanied by myriad cookbooks, blogs, and many brands producing pressure cookers. One of the hallmarks of pressure cookers is that they prepare food very quickly at a high temperature and pressure while generating steam. The ability to denature the proteins of large, thick cuts of meat in short durations is reminiscent of an autoclave, which sterilizes items at about 121°C and 15 PSI of pressure using steam generated from inside the machine or fed from a boiler. Many research groups including our own have used pressure cookers to prepare sterile media in the field, but these devices were designed such that the pressurizing compartment sat atop a separate heating element. Though reliable, they were old, occasionally broken, and required two pieces of equipment instead of one when space was at a premium. Previous work has indicated that stove-top pressure cookers can sterilize basic medical supplies, but the range of sterilization utility was not explored [22, 23]. With the surge in use of these redesigned pressure cookers in the public sector, the low price, and the ready availability, we sought to determine whether modern pressure cookers could properly sterilize items common to microbiology and other biological sciences.

An internet search indicates that the Instant Pot brand produces the most popular and most highly advertised pressure cookers. To select the pressure cooker brands used in this study, an assessment of Amazon.com reviews was undertaken to find the top three highest rated brands that produce pressure cookers similar to Instant Pot based on their consistently positive user feedback regarding dependability and durability. Ultimately, Instant Pot, GoWISE, COSORI, and Gourmia pressure cookers were chosen for study (Table 2). The 8-quart model from each brand was procured because this size was common to all brands, large enough to accommodate at least 1 L of liquid in laboratory glassware, and were priced around $100, which would make them generally accessible to many individuals. Once all four models were acquired, it was determined that a maximum of 1.5 L of liquid (split between one 1.5 L beaker holding 1 L of liquid and two 0.5 L beakers holding 0.25 L of liquid each) could fit comfortably in the metal insert within the pressure cookers without bubbling over and losing some of the contents during sterilization. To determine whether pressure cookers could sterilize microbial culture media, the minimum amount of time necessary to reliably sterilize 1.5 L of nutrient broth (NB) was determined (Table 3). The nutrient broth was prepared en masse on the laboratory bench, portioned out into the three beakers for a total of 1.5 L in each pressure cooker, and sterilized in five-minute intervals from 5–30 mins using the manual setting at the highest setting. It was found that 15 mins was the minimum time required to consistently sterilize 1.5 L of NB in all of the pressure cookers and as evidenced by a lack of microbial growth when incubated at 30°C for one week (Table 3). Similarly, when agar was added to the medium to pour plates, 15 mins was the minimum time required to consistently sterilize 1.5 L of NA plates. Less time in the pressure cookers or no sterilization resulted in turbid growth in NB and colonies on NA plates within 1–3 days of incubation at 30°C. These results indicate that pressure cookers provide sufficient heat and pressure to reliably sterilize 1.5 L of microbiological growth medium for laboratory use.

**Table 3:** Laboratory items sterilized in this study, the growth conditions used to assess sterility, and the time required to sterilize each item in all pressure cookers tested.

Item to be sterilized	Media used to assess sterilization	Growth temperature	Minimum sterilization time (mins)
Nutrient broth	-	30°C	15
Nutrient agar	-	30°C	15
Soil water	NA and SDA	30°C	45
15-cm Mall probe and seekers	NA and SDA	30°C	15
15-cm reagent digger spatulas	NA and SDA	30°C	15
Dissection scissors	NA and SDA	30°C	30

Abbreviations: NA, nutrient agar; SDA, Sabouraud dextrose agar

This initial test is a simple condition because growth medium is generally homogenous and is unlikely to contain a large microorganismal load prior to sterilization, though enough to determine whether sterilization occurred. To assess the ability of pressure cookers to sterilize heterogeneous environmental samples, soil water was prepared and the time necessary to inactivate the microbes was determined. In this experiment, soil water was created by resuspending 20 g/L of soil in MilliQ water and allowing it to settle to remove large particulates, which created a suspension of both small soil particles and the microbes present in the soil. The soil water was sterilized in 15 min intervals from 15–60 mins in each of the pressure cookers in triplicate as described above. Sterilization for 45 mins was necessary to inactivate the microbes in the soil water, which included 3 x 10^4^ CFU/mL on NA and 3.7 x 10^3^ CFU/mL on SDA, such that no growth was observed on either NA or SDA plates following one week of growth. This indicates that pressure cookers are capable of sterilizing liquid environmental samples containing particulates and harboring an uncharacterized microbial community. We infer that pressure cookers would generally be well suited to inactivate the microbes in liquids utilized in the laboratory and sterilize them for use and study.

### Pressure cookers sterilize laboratory items

The many recipes available online to create soups and stews in pressure cookers indicate that they are well suited to sterilize liquid. While sterile liquid is an integral part of most laboratories, sterile metal objects are often equally important for continued research. To determine whether metal laboratory instruments could be sterilized by pressure cookers, three common implements were dipped in soil water to be certain that they carried microbes and the time required to sterilize the instruments was determined. The instruments were chosen for their ubiquity and difference in shape: 15-cm Mall probe and seekers (hereafter probes), 15-cm reagent digger spatulas (hereafter spatulas), and dissection scissors. Probes are made of thick metal while spatulas are thin, and scissors have two pieces that steam must penetrate in between to sterilize. Separate trials were conducted with the items wrapped in aluminum foil and unwrapped in either glass culture tubes or beakers and all were sterilized in 15 min intervals from 15–60 mins in each of the pressure cookers in triplicate. Once sterilized, the instruments were submerged in either NB or SDA for one week to demonstrate that all of the microbes capable of growing in these media were inactivated. Sterilization for 15 mins was necessary to inactivate the microbes on the spatula and probe, but 30 mins in the pressure cookers was required to sterilize the scissors (Table 3). As the soil water left on the items touched the culture tubes, and they were occasionally manipulated outside of the hood, we infer that the culture tubes were also sterilized in these trials because no growth was evident following incubation with growth medium. Items that were sterilized for less time or not run through the pressure cookers showed growth in both media types within three days of incubation. This indicates that pressure cookers could be used to sterilize metal instruments and glassware for laboratory manipulation.

### High titers of bacteria and fungi are inactivated with pressure cookers

**Fig 1:**
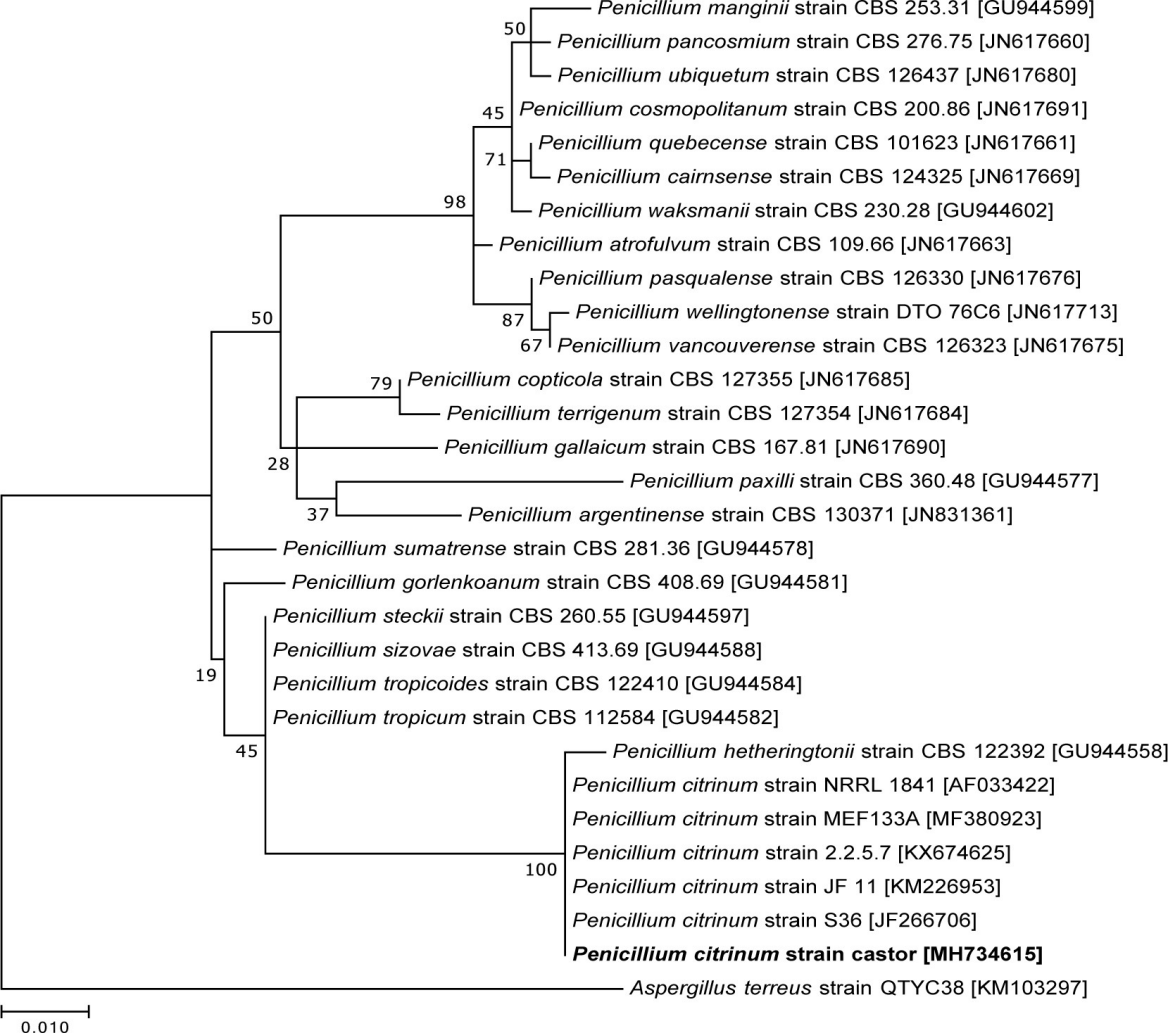
Dendrogram displaying the phylogenetic relationship between *Penicillium citrinum* strain castor and other *Penicillium* strains. Relatedness was inferred using the Maximum Likelihood method and 1000 bootstrap replicates were performed. Next to each branch, the percentage of trees with the associated taxa is shown. The scale represents 0.01 substitutions per site. *Aspergillus terreus* strain QTYC38 was chosen as an outgroup. The strain identified in this study is in bold and GenBank accession numbers are in brackets next to the strain names.

While the experimentation thus far has sterilized the largest volumes that will fit in the pressure cookers tested, none of the manipulations has utilized a high concentration of microorganisms. It is common to culture 10^7^ CFU/mL of pathogenic microbes in the laboratory, or have this concentration of pathogens on disease samples from the environment, and then need to dispose of them in a manner that will not harm the researcher or the populace. To determine whether pressure cookers can inactivate high titers of microbes, various bacteria and fungi were cultured in liquid media and the time required to inactivate them was determined. Microorganisms with diverse physiologies were selected including Gram positive and negative, rod, cocci, and vibrio bacteria, and filamentous and unicellular fungi, which included several medically relevant pathogens. While most strains tested were laboratory acclimated type strains, the filamentous fungus tested was a laboratory isolate, which clustered *Penicillium citrinum* by phylogenetic analysis of 18S rRNA locus sequences and named strain castor (Fig 1 and S1 Fig). Incorporating a laboratory isolate into the analysis provided a realistic condition that researchers could encounter; the disposal of a culture derived from an environmental isolate. Large cultures of the desired microbe were prepared and the CFU/mL was determined by dilution series (Table 1). The cultures were run in 15 min intervals in 1.5 L aliquots from 15–75 mins in each of the pressure cookers in triplicate as described above. Culture inactivation was determined by plating 1 mL of the sterilized culture on the corresponding growth medium in triplicate and visually assessing colony formation (S2 Fig). Sterilization times of at least 15 mins were required to inhibit growth of the bacterial strains tested while 60 mins was necessary to inhibit fungal growth. These results indicate that the pressure cookers tested are able to inactivate up to 10^10^ CFU/mL of bacteria and roughly 10^7^ CFU/mL of fungi.

### Instant Pot pressure cooker passes the spore sterilization test

**Fig 2:**
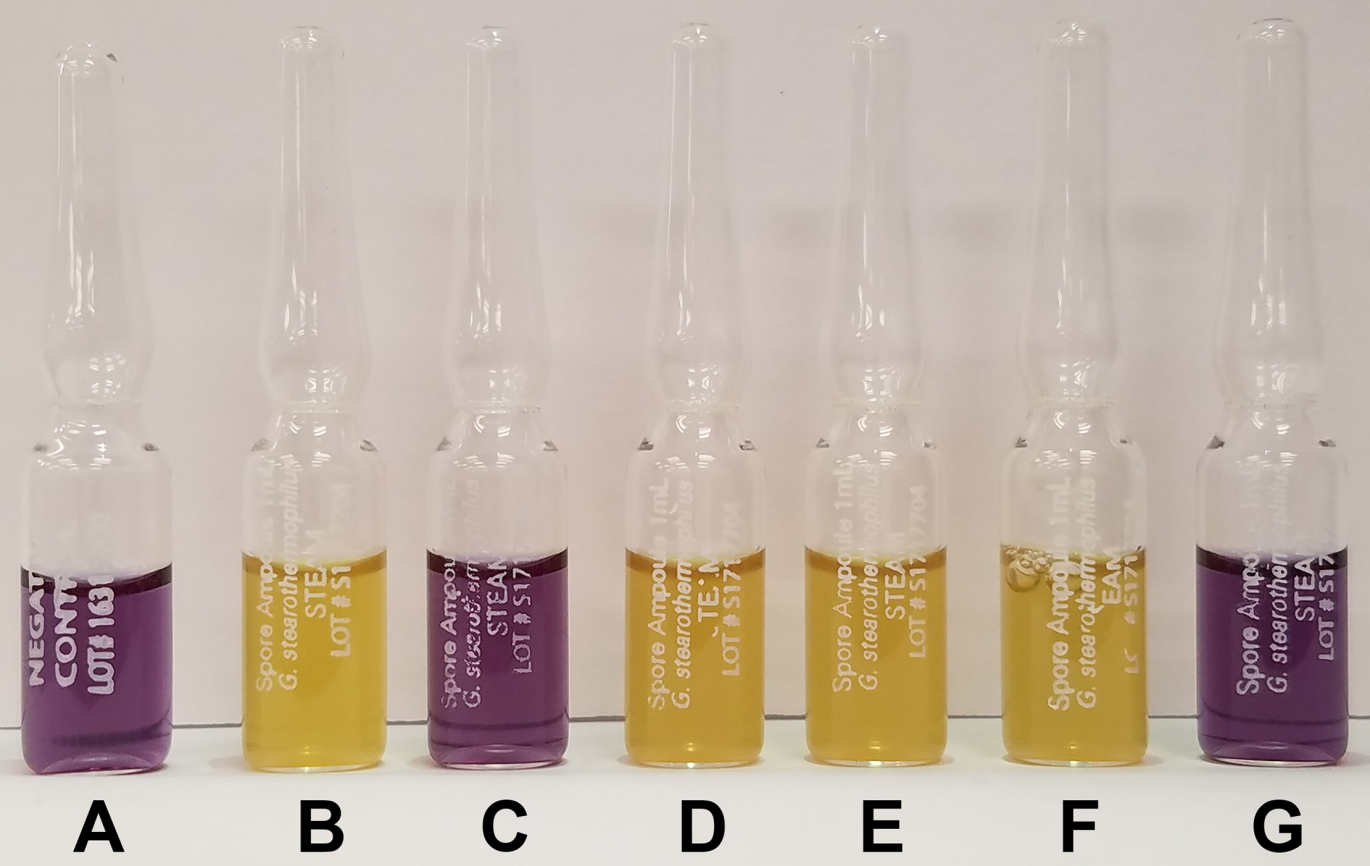
The Instant Pot pressure cooker was able to inactivate *Geobacillus stearothermophilus* spore ampoule biological tests. The spore ampoules were run for 60 min in either an autoclave (C) and 150 min in the COSORI (D), GoWise (E), Gourmia (F), or Instant Pot pressure cooker. A negative control ampoule (A) and positive control ampoule that was not sterilized (B) were included. All ampoules were incubated at 60°C for 48 h and assessed visually for color change from purple to yellow and increased turbidity. The writing on the ampoules is presented in S1 Table.

Regular maintenance and testing are required to certify the functionality of autoclaves and other steam sterilization devices used in industry, medicine, and academe. Of the many available products to verify sterilization, one common method involves the inactivation of *Geobacillus stearothermophilus* spores, which have long been known to be especially heat resistant [6]. These tests generally stipulate that a spore ampoule be placed in the most difficult location in the load for steam to reach, run the standard cycle, and then either incubate the ampoule and record any color or turbidity changes or spread the contents on a plate and assess colony formation. Should the ampoule remain unchanged and transparent, or the plate fail to grow colonies, the spores have been inactivated and the sterilization device is working properly. Guidelines for sterilizer function regarding the time, temperature, and pressure necessary to inactivate *G*. *stearothermophilus* spores have been determined by the FDA, USP, and ISO 11138–3. If a sterilization device is capable of passing a spore test, it implies that all other less heat resistant organisms can be inactivated and that the device is sterilizing the contents to a level that would be acceptable for laboratory use. To verify that commercially available pressure cookers are capable of sterilizing laboratory items to the same level as an autoclave, the inactivation of ampoules containing 10^6^ *G*. *stearothermophilus* spores was assessed. Within these pressure cookers, the most difficult place to sterilize is presumably at the bottom of the 1 L of liquid in the 1.5 L beaker. Each pressure cooker was loaded with all three beakers containing 1.5 L of water and the spore ampoule was weighted to the bottom via attachment to a metal washer. The spore ampoules were run in 30 min intervals from 30–300 mins in each of the pressure cookers in triplicate as described above and allowed to incubate at 60°C for 48 h. Positive spore tests remained purple and transparent, which indicated that the spores were inactivated, while negative tests turned yellow and displayed turbid growth. A sterilization time of at least 150 mins was required for the Instant Pot to inactivate the spores (Fig 2), which appeared similar in color and opacity to a spore ampoule sterilized in an autoclave and the negative control. None of the other pressure cookers inactivated the spores even with sterilization times of up to 300 mins, which yielded unsterilized spore ampoules visually identical to a positive control. These data indicated that the Instant Pot pressure cooker could sterilize spores to the level of an autoclave, and we infer that it would be capable of inactivating all other less heat resistant organisms to properly sterilize laboratory items for research.

## Discussion

One of the hallmarks of the scientific endeavor involves the generation of reproducible data that withstands scrutiny over time. To manipulate axenic cultures and consistently maintain their purity, it is necessary to remove or inactivate all other microbes capable of contaminating the desired research organism. Without sterile consumables in a prepared workspace, unwanted organisms may be introduced into experiments altering results and invalidating the research or compromising a patient. While we may take this for granted in much of the USA, such sterilized items or surfaces are not available to many in the developing world, in the field, and following natural disasters that destroy infrastructure. Alternative methods for the sterile preparation of items is immediately necessary to democratize science and medicine, which could allow those under financial constraint improved access to sterilization techniques. In this work, we assessed the viability of four brands of 8-quart pressure cooker to sterilize biological consumables and instrumentation. All four brands inactivated the microbes contaminating microbiological culture media and on metal implements within 15 mins and 30 mins of run time, respectively. The pressure cookers tested also inactivated high titers of bacteria and fungi, some of which are medically relevant strains, within 15 mins and 60 mins of run time, respectively. Unlike the other brands tested, the Instant Pot was capable of inactivating 10^6^ *G*. *stearothermophilus* spores, which indicates that it can sterilize items to the level generally deemed acceptable for laboratory autoclaves. These data demonstrate that the pressure cookers tested are a viable alternative for steam sterilizing laboratory items when an autoclave is unavailable.

In this work, only 15 mins of time in the pressure cookers was necessary to inactivate the microbes in growth media such that no growth was observed. High titer cultures of fungi required 60 mins for inactivation, which was the longest run time for live organisms tested. In these experiments, the absence of growth following prolonged incubation was used as the indicator of microbial inactivation. In contrast, 150 mins of run time was needed to consistently inactivate *G*. *stearothermophilus* spores, though half of the spore ampoules tested were inactivated after 120 mins in the Instant Pot. The increased tolerance of spores to heat and pressure is well known so the difference in inactivation time between vegetative cells and spores is not unexpected. None of the conditions that included unknown microbial constituents (soil water and media) required more than 45 mins for inactivation. This indicates that there were potentially fewer spores in these preparations, the spores present were more easily inactivated than *G*. *stearothermophilus* spores, or that spores capable of withstanding pressure cooking were present but could not germinate in the media provided. No matter the reason, no colonies were ever detected after trials run for the stated durations, which is sufficient to consider the microbes inactivated and the preparation usable for laboratory manipulation. Indeed, this is the same standard to which autoclaves are held; resistant spores may be present in/on autoclaved items, but they fail to germinate so the items are considered sterile.

All four pressure cookers tested were able to inactivate the microbes present in all of the trials except for the inactivation of *G*. *stearothermophilus* spores, in which only the Instant Pot was able to produce steam, pressure, and heat to the level necessary to achieve sterilizing conditions. When initially purchased near the holidays, all of the pressure cookers retailed for roughly the same price, however, a recent price check indicated that the Instant Pot is a much more expensive model (Table 2). It is possible that the Instant Pot is constructed with higher quality materials, which allow it to reach and sustain a higher internal working pressure and temperature and makes it more expensive than the other models. The locking mechanisms and O-rings holding the lids in place are differently constructed between the models and this may contribute to the ability to maintain a higher pressure. We also observed that the Instant Pot took longer to pressurize than the other models, which could indicate that more steam is generated during this protracted pressurization. It is also possible that the heating elements differ between the pressure cookers, and this could account for differences in the ability to inactivate highly resistant spores in a manner similar to an autoclave.

The systematic characterization and validation of common pressure cookers as acceptable laboratory sterilization devices provides the impetus for their use in a number of situations. The use of single piece construction pressure cookers as sterilization devices could be helpful to researchers conducting field studies because these instruments are small, fairly lightweight, and are readily shipped when load size and weight are a concern. Our previous work used a pressure cooker to prepare sterile growth medium while conducting research at Palmyra Atoll in the Pacific Line Islands, which facilitated the isolation and testing of a bacterial coral pathogen [24]. Other groups could use these on scientific cruises, in remote locations, and when all items must be backpacked into sites. As the pressure cookers tested are less than $100 when purchased during non-holiday times, they could also be beneficial for classroom activities and demonstrations in middle and high schools. Budget constraints in the United States public school system does not generally allow for the purchase of large expensive instrumentation, but the incorporation of small inexpensive instruments into classrooms can provide additional, previously unattainable hands-on opportunities for student learning and engagement. Furthermore, these pressure cookers could expand research capabilities in underfunded laboratories in developing countries where large instrumentation like autoclaves is prohibitively expensive. This work demonstrates that pressure cookers can sterilize common laboratory items and can be used to improve research and teaching capacities in numerous situations.

## Supporting information

S1 Fig ITS sequence alignment. Alignment of the ITS sequences from the 30 strains used to create the dendrogram in Fig 1. The strain identified in this study is in bold and conserved bases are denoted by an asterisk below the alignment. The genus denoted by A. is *Aspergillus* and by P. is *Penicillium*.
(PDF)

S2 Fig Cultures inactivation in pressure cookers. Representative culture before (left panels) and after (right panels) pressure cooker sterilization. *Bacillus subtilis* was grown in nutrient broth overnight with aeration and the culture is visibly turbid (upper left panel), and produced a lawn of growth when spread on a nutrient agar plate (lower left panel). After pressure cooker sterilization, the broth was a caramel color with increased sedimentation at the bottom of the beaker and a nearly transparent layer at the top (upper right panel). Complete optical transparency was not achieved following sterilization in a pressure cooker or an autoclave likely because the cytoplasmic contents of lysed cells increased the opacity of the broth. No growth was visible when the pressure cooker-sterilized culture was spread on a nutrient agar plate (bottom right panel).
(PDF)

S1 Table Spore ampoule text. Transcription of the text on the ampoules in Fig 2.
(DOCX)
