# Supplementary material for: Assessment and verification of commercially available pressure cookers for laboratory sterilization
Source: PLoS One. 2018 Dec 11;13(12):e0208769. doi: 10.1371/journal.pone.0208769 (PMC6289433; doi:10.1371/journal.pone.0208769)
Supplement: S1 Fig — Alignment of the ITS sequences from the 30 strains used to create the dendrogram in Fig 1. The strain identified in this study is in bold and conserved bases are denoted by an asterisk below the alignment. The genus denoted by A. is Aspergillus and by P. is Penicillium. (PDF) [file pone.0208769.s001.pdf]

```
A. terreus QTYC38              -GACTGAGGGGCCTCTGGGTC-CACCTCCCACCCGTGTCTATC-GTACCTTGTTGCTTCG   57
P. paxilli CBS 360.38          -GAGTGAGGGCCCTCTGGGTCCAACCTCCCACCCGTGTTTAACTGTACCTTGTTGCTTCG   59
P. argentinense CBS 130371     GAGTGAGGGCCCCTCCGGGTCCAACCTCCCACCCGTGTTTAACGAA-CCTTGTTGCTTCG   59
P. pasqualense CBS 126330      GAGTGAGGGCCCCTCGGGGTCCAACCTCCCACCCGTGTTTAACGAA-CCTTGTTGCTTCG   59
P. wellingtonense DTO 76C6     GAGTGAGGGCCCCTCGGGGTCCAACCTCCCACCCGTGTTTAACGAA-CCTTGTTGCTTCG   59
P. vancouverense CBS 126323    GAGTGAGGGCCCCTCGGGGTCCAACCTCCCACCCGTGTTTAACGAA-CCTTGTTGCTTCG   59
P. ubiqetum CBS 126437         GAGTGAGGGCCCCTCGGGGTCCAACCTCCCACCCGTGTTTAACGAA-CCTTGTTGCTTCG   59
P. manginii CBS 253.31         GAGTGAGGGCCCCTCGGGGTCCAACCTCCCACCCGTGTTTAACGAA-CCGTGTTGCTTCG   59
P. pancosmium CBS 276.75       GAGTGAGGGCCCCTCGGGGTCCAACCTCCCACCCGTGTTTAACGTA-CCTTGTTGCTTCG   59
P. atrofulvum CBS 109.66       GAGTGAGGGCCCCTCGGGGTCCAACCTCCCACCCGTGTTTAACGAA-CCTTGTTGCTTCG   59
P. quebecense CBS 101623       GAGTGAGGGCCCCTCGGGGTCCAACCTCCCACCCGTGTTTAACGAA-CCTTGTTGCTTCG   59
P. cairnsence CBS 124325       GAGTGAGGGCCCCTCGGGGTCCAACCTCCCACCCGTGTTTAACGAA-CCTTGTTGCTTCG   59
P. waksmanii CBS 230.28        GAGTGAGGGCCCCTCGGGGTCCAACCTCCCACCCGTGTTTAACGAA-CCTTGTTGCTTCG   59
P. cosmopolitanum CBS 200.86   GAGTGAGGGCCCCTCGGGGTCCAACCTCCCACCCGTGTTTAACGAA-CCTTGTTGCTTCG   59
P. sumartrense CBS 281.36      GAGTGAGGGCCCCTCGGGGTCCAACCTCCCACCCGTGTTTAACGAACCCTTGTTGCTTCG   60
P. gallaicum CBS 167.81        -GAGTGAGGGCCCTCCGGGTCCAACCTCCCACCCGTGTTTA-ACGAACCTTGTTGCTTCG   58
P. copticola CBS 127355        -GAGTGAGGGCCCTCTGGGTCCAACCTCCCACCCGTGTTTT-CCGAACCCTGTTGCTTCG   58
P. terrigenum CBS 127354       -GAGTGAGGGCCCTCTGGGTCCAACCTCCCACCCGTGTTTT-CCGAACCCTGTTGCTTCG   58
P. hetheringtonii CBS 122392   GAGTGCGGGCCCCTCGGGGCCCAACCTCCCACCCGTGTTGCCCGAACCTATG--------   52
P. citrinum NRRL 1841          GAGTGCGGGCCCCTCGGGGCCCAACCTCCCACCCGTGTTGCCCGAACCTATG--------   52
P. citrinum MEF133A            GAGTGCGGGCCCCTCGGGGCCCAACCTCCCACCCGTGTTGCCCGAACCTATG--------   52
P. citrinum 2.2.5.7            GAGTGCGGGCCCCTCGGGGCCCAACCTCCCACCCGTGTTGCCCGAACCTATG--------   52
P. citrinum JF 11              GAGTGCGGGCCCCTCGGGGCCCAACCTCCCACCCGTGTTGCCCGAACCTATG--------   52
P. citrinum S36                GAGTGCGGGCCCCTCGGGGCCCAACCTCCCACCCGTGTTGCCCGAACCTATG--------   52
P. citrinum castor             GAGTGCGGGCCCCTCGGGGCCCAACCTCCCACCCGTGTTGCCCGAACCTATG--------   52
P. gorlenkoanum CBS 408.69     -GAGTGAGGGCCCTCTGGGTCCAACCTCCCACCCGTGTTTTAACGAACCTTTGTTGCTTC   59
P. steckii CBS 260.55          -GAGTGAGGGCCCTCTGGGTCCAACCTCCCACCCGTGTTGCACGAACCTGTGTTGCTTCG   59
P. tropicum CBS 112584         -GAGTGAGGGCCCTCTGGGTCCAACCTCCCACCCGTGTTGCACGAACCTGTGTTGCTTCG   59
P. tropicoides CBS 122410      -GAGTGAGGGCCCTCTGGGTCCAACCTCCCACCCGTGTTGCACGAACCTGTGTTGCTTCG   59
P. sizovae CBS 413.69          -GAGTGAGGGCCCTCTGGGTCCAACCTCCCACCCGTGTTGCACGAACCTGTGTTGCTTCG   59
                                      **  **** *** *  ***************         *  *

A. terreus QTYC38              GCGGGCCCGCCGTTTCGACGGCCGCCGGGGAGGCCTCGCGCCCCCGGGCCCGCGCCCGCC   117
P. paxilli CBS 360.38          GCGGGCCCGCCTCACG----GCCGCCGGGGGGCTCTCCTGCCCCCGGGCCCGCGCCCGCC   115
P. argentinense CBS 130371     GCGGGCCCGCCGTATG----GCCGCCGGGGGG--CACCCGCCCCCGGGCCCGCGCCCGCC   113
P. pasqualense CBS 126330      GCGAGCCCGCCTCACG----GCCGCCGGGGGG--CATCTGCCCCCGGGCCCGCGCCCGCC   113
P. wellingtonense DTO 76C6     GCGAGCCCGCCTCACG----GCCGCCGGGGGG--CATCTGCCCCCGGGCCCGCGCCCGCC   113
P. vancouverense CBS 126323    GCGAGCCCGCCTCACG----GCCGCCGGGGGG--CATCTGCCCCCGGGCCCGCGCCCGCC   113
P. ubiqetum CBS 126437         GCGGGCCCGCCTCACG----GCCGCCGGGGGG--CATCCGCCCCCGGGCCCGCGCCCGCC   113
P. manginii CBS 253.31         GCGGGCCCGCCTCACG----GCCGCCGGGGGG--CATCCGCCCCCGGGCCCGCGCCCGCC   113
P. pancosmium CBS 276.75       GCGGGCCCGCCTCACG----GCCGCCGGGGGG--CATCCGCCCCCGGGCCCGCGCCCGCC   113
P. atrofulvum CBS 109.66       GCGGGCCCGCCTCACG----GCCGCCGGGGGG--CATCTGCCCCCGGGCCCGCGCCCGCC   113
P. quebecense CBS 101623       GCGGGCCCGCCTCACG----GCCGCCGGGGGG--CATCTGCCCCCGGGTCCGCGCCCGCC   113
P. cairnsence CBS 124325       GCGGGCCCGCCTCACG----GCCGCCGGGGGG--CATCTGCCCCCGGGTCCGCGCCCGCC   113
P. waksmanii CBS 230.28        GCGGGCCCGCCTCACG----GCCGCCGGGGGG--CATCCGCCCCCGGGCCCGCGCCCGCC   113
P. cosmopolitanum CBS 200.86   GCGGGCCCGCCTCACG----GCCGCCGGGGGG--CATCTGCCCCCGGGCCCGCGCCCGCC   113
P. sumartrense CBS 281.36      GCGGGCCCGCCTCACG----GCCGCCGGGGGG--CTCCTGCCCCCGGGCCCGCGCCCGCC   114
P. gallaicum CBS 167.81        GCGGGCCCGCCTCACG----GCCGCCGGGGGG--CCTCTGCCCCCGGGCCCGCGCCCGCC   112
P. copticola CBS 127355        GCGGGCCCGCCTCACG----GCCGCCGGGGGG--CTTCCGCCCCCGGGCCCGCGCCCGCC   112
P. terrigenum CBS 127354       GCGGGCCCGCCTCACG----GCCGCCGGGGGG--CTTCTGCCCCCGGGCCCGCGCCCGCC   112
P. hetheringtonii CBS 122392   ----------------------------------TTGCCTCGGCGGGCCCCGCGCCCGCC   78
P. citrinum NRRL 1841          ----------------------------------TTGCCTCGGCGGGCCCCGCGCCCGCC   78
P. citrinum MEF133A            ----------------------------------TTGCCTCGGCGGGCCCCGCGCCCGCC   78
P. citrinum 2.2.5.7            ----------------------------------TTGCCTCGGCGGGCCCCGCGCCCGCC   78
P. citrinum JF 11              ----------------------------------TTGCCTCGGCGGGCCCCGCGCCCGCC   78
P. citrinum S36                ----------------------------------TTGCCTCGGCGGGCCCCGCGCCCGCC   78
P. citrinum castor             ----------------------------------TTGCCTCGGCGGGCCCCGCGCCCGCC   78
P. gorlenkoanum CBS 408.69     GGCGGGCCCGCCGCCA----GGCCGCCGGGGGGCATCCGCCCCCGGGCCCGCGCCCGCCG   115
P. steckii CBS 260.55          GCGGGCCCGCCGCCA-----GGCCGCCGGGGGGCATCCGCCCCCGGGCCCGCGCCCGCCG   114
P. tropicum CBS 112584         GCGGGCCCGCCGCCTA----GGCCGCCGGGGGGCATCCGCCCCCGGGCCCGCGCCCGCCG   115
P. tropicoides CBS 122410      GCGGGCCCGCCGCCTA----GGCCGCCGGGGGGCATCCGCCCCCGGGCCCGCGCCCGCCG   115
P. sizovae CBS 413.69          GCGGGCCCGCCGCCTA----GGCCGCCGGGGGGCATCCGCCCCCGGGCCCGCGCCCGCCG   115
                                                                       *  * **  *    **  *
```

```
A. terreus QTYC38                GAAGACCCCAACATGAACTCTGTTCTGAAAGTATGCAGTCTGAGTTGATTATCATAATCA   177
P. paxilli CBS 360.38            GAAGACA----CCTGTGAACGCTGTCTGAAGTATGCAGTCTGAGAAAACTAGCTAAATTA   171
P. argentinense CBS 130371       GAAGACA----CCTGTGAACGCTGTCTGAAGTTG-CAGTCTGAGACAACTAGCTAAATTA   168
P. pasqualense CBS 126330        GAAGCCA----CCTGTGAAC-TCGTCTGAAGTAT-GCAGTCTGAGACAATTATTAAATTA   167
P. wellingtonense DTO 76C6       GAAGCCA----CCTGTGAAC-TCGTCTGAAGTAT-GTAGTCTGAGACAATTATTAAATTA   167
P. vancouverense CBS 126323      GAAGCCA----CCTGTGAAC-TCGTCTGAAGTAT-GTAGTCTGAGACAATTATTAAATTA   167
P. ubiqetum CBS 126437           GAAGCCA----CCTGTGAACGCTGTCTGAAGTAT-GCAGTCTGAGACAATTATTAAATTA   168
P. manginii CBS 253.31           GAAGCCC----CCTGTGAACGCTGTCTGAAGTAT-GCAGTCTGAGACAATTATTCAATTA   168
P. pancosmium CBS 276.75         GAAGCCA----CCTGTGAACGCTGTCTGAAGTAT-GCAGTCTGAGACAATTATTAAATTA   168
P. atrofulvum CBS 109.66         GAAGCCA----CCTGTGAACGCTGTCTGAAGTAT-GCAGTCTGAGACAATTATTAAATTA   168
P. quebecense CBS 101623         GAAGCCA----CCTGTGAACTCTGTCTGAAGTAT-GCAGTCTGAGACAATTATTAAATTA   168
P. cairnsence CBS 124325         GAAGCCA----CCTGTGAACTCTGTCTGAAGTAT-GCAGTCTGAGACAATTATTAAATTA   168
P. waksmanii CBS 230.28          GAAGCCA----CCTGTGAACGCTGTCTGAAGTAT-GCAGTCTGAGACAATTATTAAATTA   168
P. cosmopolitanum CBS 200.86     GAAGCCA----CCTGTGAACGCTGTCTGAAGTAT-GCAGTCTGAGACAATTATTAAATTA   168
P. sumartrense CBS 281.36        GAAGCCC----CCCCTGAACGCTGTCTGAAG-TT-GCAGTCTGAGAAACTAGCTAAATTA   168
P. gallaicum CBS 167.81          GAAGACA----CCTGTGAACGCTGTCTGAAG-TT-GCAGTCTGAGACACTAGCTAAATCA   166
P. copticola CBS 127355          GAAGACA----CCTGTGAACGCTGTCTGAAG-TT-GCAGTCTGAGAAACTAGCTAAATTA   166
P. terrigenum CBS 127354         GAAGACA----CCTGTGAACGCTGTCTGAAG-TT-GCAGTCTGAGAAACTAGCTAAATTA   166
P. hetheringtonii CBS 122392     GACGGCC----CCCCTGAACGCTGTCTGAAGTTG-CAGTCTGAGACCTATAACGAAATTA   133
P. citrinum NRRL 1841            GACGGCC----CCCCTGAACGCTGTCTGAAGTTG-CAGTCTGAGACCTATAACGAAATTA   133
P. citrinum MEF133A              GACGGCC----CCCCTGAACGCTGTCTGAAGTTG-CAGTCTGAGACCTATAACGAAATTA   133
P. citrinum 2.2.5.7              GACGGCC----CCCCTGAACGCTGTCTGAAGTTG-CAGTCTGAGACCTATAACGAAATTA   133
P. citrinum JF 11                GACGGCC----CCCCTGAACGCTGTCTGAAGTTG-CAGTCTGAGACCTATAACGAAATTA   133
P. citrinum S36                  GACGGCC----CCCCTGAACGCTGTCTGAAGTTG-CAGTCTGAGACCTATAACGAAATTA   133
P. citrinum castor               GACGGCC----CCCCTGAACGCTGTCTGAAGTTG-CAGTCTGAGACCTATAACGAAATTA   133
P. gorlenkoanum CBS 408.69       AAGCCCC----CCTCTGAACGCTGTCTGAAGTTG-CAGTCTGAGACAACTAGCTAAATTA   170
P. steckii CBS 260.55            AAGCCCC----CCTCTGAACGCTGTCTGAAGTTG-CAGTCTGAGACAACTAGCTAAATTA   169
P. tropicum CBS 112584           AAGCCCC----CCTCTGAACGCTGTCTGAAGTTG-CAGTCTGAGACAACTAGCTAAATTA   170
P. tropicoides CBS 122410        AAGCCCC----CCTCTGAACGCTGTCTGAAGTTG-CAGTCTGAGACAACTAGCTAAATTA   170
P. sizovae CBS 413.69            AAGCCCC----CCTCTGAACGCTGTCTGAAGTTG-CAGTCTGAGAA-ACTAGCTAAATTA   169
                                  *   *     *       *        ***                  *     *** *

A. terreus QTYC38                GTTAAAACTTTCAACAACGGATCTCTTGGTTCCGGCATCGATGAAGAACGCAGCGAAATG   237
P. paxilli CBS 360.38            GTTAAAACTTTCAACAACGGATCTCTTGGTTCCGGCATCGATGAAGAACGCAGCGAAATG   231
P. argentinense CBS 130371       GTTAAAACTTTCAACAACGGATCTCTTGGTTCCGGCATCGATGAAGAACGCAGCGAAATG   228
P. pasqualense CBS 126330        ATTAAAACTTTCAACAACGGATCTCTTGGTTCCGGCATCGATGAAGAACGCAGCGAAATG   227
P. wellingtonense DTO 76C6       ATTAAAACTTTCAACAACGGATCTCTTGGTTCCGGCATCGATGAAGAACGCAGCGAAATG   227
P. vancouverense CBS 126323      ATTAAAACTTTCAACAACGGATCTCTTGGTTCCGGCATCGATGAAGAACGCAGCGAAATG   227
P. ubiqetum CBS 126437           ATTAAAACTTTCAACAACGGATCTCTTGGTTCCGGCATCGATGAAGAACGCAGCGAAATG   228
P. manginii CBS 253.31           ATTAAAACTTTCAACAACGGATCTCTTGGTTCCGGCATCGATGAAGAACGCAGCGAAATG   228
P. pancosmium CBS 276.75         ATTAAAACTTTCAACAACGGATCTCTTGGTTCCGGCATCGATGAAGAACGCAGCGAAATG   228
P. atrofulvum CBS 109.66         ATTAAAACTTTCAACAACGGATCTCTTGGTTCCGGCATCGATGAAGAACGCAGCGAAATG   228
P. quebecense CBS 101623         ATTAAAACTTTCAACAACGGATCTCTTGGTTCCGGCATCGATGAAGAACGCAGCGAAATG   228
P. cairnsence CBS 124325         ATTAAAACTTTCAACAACGGATCTCTTGGTTCCGGCATCGATGAAGAACGCAGCGAAATG   228
P. waksmanii CBS 230.28          ATTAAAACTTTCAACAACGGATCTCTTGGTTCCGGCATCGATGAAGAACGCAGCGAAATG   228
P. cosmopolitanum CBS 200.86     ATTAAAACTTTCAACAACGGATCTCTTGGTTCCGGCATCGATGAAGAACGCAGCGAAATG   228
P. sumartrense CBS 281.36        GTTAAAACTTTCAACAACGGATCTCTTGGTTCCGGCATCGATGAAGAACGCAGCGAAATG   228
P. gallaicum CBS 167.81          GTTAAAACTTTCAACAACGGATCTCTTGGTTCCGGCATCGATGAAGAACGCAGCGAAATG   226
P. copticola CBS 127355          GTTAAAACTTTCAACAACGGATCTCTTGGTTCCGGCATCGATGAAGAACGCAGCGAAATG   226
P. terrigenum CBS 127354         GTTAAAACTTTCAACAACGGATCTCTTGGTTCCGGCATCGATGAAGAACGCAGCGAAATG   226
P. hetheringtonii CBS 122392     ATTAAAACTTTCAACAACGGATCTCTTGGTTCCGGCATCGATGAAGAACGCAGCGAAATG   193
P. citrinum NRRL 1841            GTTAAAACTTTCAACAACGGATCTCTTGGTTCCGGCATCGATGAAGAACGCAGCGAAATG   193
P. citrinum MEF133A              GTTAAAACTTTCAACAACGGATCTCTTGGTTCCGGCATCGATGAAGAACGCAGCGAAATG   193
P. citrinum 2.2.5.7              GTTAAAACTTTCAACAACGGATCTCTTGGTTCCGGCATCGATGAAGAACGCAGCGAAATG   193
P. citrinum JF 11                GTTAAAACTTTCAACAACGGATCTCTTGGTTCCGGCATCGATGAAGAACGCAGCGAAATG   193
P. citrinum S36                  GTTAAAACTTTCAACAACGGATCTCTTGGTTCCGGCATCGATGAAGAACGCAGCGAAATG   193
P. citrinum castor               GTTAAAACTTTCAACAACGGATCTCTTGGTTCCGGCATCGATGAAGAACGCAGCGAAATG   193
P. gorlenkoanum CBS 408.69       GTTAAAACTTTCAACAACGGATCTCTTGGTTCCGGCATCGATGAAGAACGCAGCGAAATG   230
P. steckii CBS 260.55            GTTAAAACTTTCAACAACGGATCTCTTGGTTCCGGCATCGATGAAGAACGCAGCGAAATG   229
P. tropicum CBS 112584           GTTAAAACTTTCAACAACGGATCTCTTGGTTCCGGCATCGATGAAGAACGCAGCGAAATG   230
P. tropicoides CBS 122410        GTTAAAACTTTCAACAACGGATCTCTTGGTTCCGGCATCGATGAAGAACGCAGCGAAATG   230
P. sizovae CBS 413.69            GTTAAAACTTTCAACAACGGATCTCTTGGTTCCGGCATCGATGAAGAACGCAGCGAAATG   229
                                  ***********************************************************
```

A. terreus QTYC38	CGATAAGTAATGTGAATTGCAGAATTCAGTGAATCATCGAGTCTTTGAACGCACATTGCG	297
P. paxilli CBS 360.38	CGATAATTAATGTGAATTGCAGAATTCAGTGAATCATCGAGTCTTTGAACGCACATTGCG	291
P. argentinense CBS 130371	CGATAATTAATGTGAATTGCAGAATTCAGTGAATCATCGAGTCTTTGAACGCACATTGCG	288
P. pasqualense CBS 126330	CGATAACTAATGTGAATTGCAGAATTCAGTGAATCATCGAGTCTTTGAACGCACATTGCG	287
P. wellingtonense DTO 76C6	CGATAACTAATGTGAATTGCAGAATTCAGTGAATCATCGAGTCTTTGAACGCACATTGCG	287
P. vancouverense CBS 126323	CGATAACTAATGTGAATTGCAGAATTCAGTGAATCATCGAGTCTTTGAACGCACATTGCG	287
P. ubiqetum CBS 126437	CGATAACTAATGTGAATTGCAGAATTCAGTGAATCATCGAGTCTTTGAACGCACATTGCG	288
P. manginii CBS 253.31	CGATAACTAATGTGAATTGCAGAATTCAGTGAATCATCGAGTCTTTGAACGCACATTGCG	288
P. pancosmium CBS 276.75	CGATAACTAATGTGAATTGCAGAATTCAGTGAATCATCGAGTCTTTGAACGCACATTGCG	288
P. atrofulvum CBS 109.66	CGATAACTAATGTGAATTGCAGAATTCAGTGAATCATCGAGTCTTTGAACGCACATTGCG	288
P. quebecense CBS 101623	CGATAACTAATGTGAATTGCAGAATTCAGTGAATCATCGAGTCTTTGAACGCACATTGCG	288
P. cairnsence CBS 124325	CGATAACTAATGTGAATTGCAGAATTCAGTGAATCATCGAGTCTTTGAACGCACATTGCG	288
P. waksmanii CBS 230.28	CGATAACTAATGTGAATTGCAGAATTCAGTGAATCATCGAGTCTTTGAACGCACATTGCG	288
P. cosmopolitanum CBS 200.86	CGATAACTAATGTGAATTGCAGAATTCAGTGAATCATCGAGTCTTTGAACGCACATTGCG	288
P. sumartrense CBS 281.36	CGATAACTAATGTGAATTGCAGAATTCAGTGAATCATCGAGTCTTTGAACGCACATTGCG	288
P. gallaicum CBS 167.81	CGATAACTAATGTGAATTGCAGAATTCAGTGAATCATCGAGTCTTTGAACGCACATTGCG	286
P. copticola CBS 127355	CGATAACTAATGTGAATTGCAGAATTCAGTGAATCATCGAGTCTTTGAACGCACATTGCG	286
P. terrigenum CBS 127354	CGATAACTAATGTGAATTGCAGAATTCAGTGAATCATCGAGTCTTTGAACGCACATTGCG	286
P. hetheringtonii CBS 122392	CGATAACTAATGTGAATTGCAGAATTCAGTGAATCATCGAGTCTTTGAACGCACATTGCG	253
P. citrinum NRRL 1841	CGATAACTAATGTGAATTGCAGAATTCAGTGAATCATCGAGTCTTTGAACGCACATTGCG	253
P. citrinum MEF133A	CGATAACTAATGTGAATTGCAGAATTCAGTGAATCATCGAGTCTTTGAACGCACATTGCG	253
P. citrinum 2.2.5.7	CGATAACTAATGTGAATTGCAGAATTCAGTGAATCATCGAGTCTTTGAACGCACATTGCG	253
P. citrinum JF 11	CGATAACTAATGTGAATTGCAGAATTCAGTGAATCATCGAGTCTTTGAACGCACATTGCG	253
P. citrinum S36	CGATAACTAATGTGAATTGCAGAATTCAGTGAATCATCGAGTCTTTGAACGCACATTGCG	253
***P. citrinum* castor**	CGATAACTAATGTGAATTGCAGAATTCAGTGAATCATCGAGTCTTTGAACGCACATTGCG	253
P. gorlenkoanum CBS 408.69	CGATAACTAATGTGAATTGCAGAATTCAGTGAATCATCGAGTCTTTGAACGCACATTGCG	290
P. steckii CBS 260.55	CGATAACTAATGTGAATTGCAGAATTCAGTGAATCATCGAGTCTTTGAACGCACATTGCG	289
P. tropicum CBS 112584	CGATAACTAATGTGAATTGCAGAATTCAGTGAATCATCGAGTCTTTGAACGCACATTGCG	290
P. tropicoides CBS 122410	CGATAACTAATGTGAATTGCAGAATTCAGTGAATCATCGAGTCTTTGAACGCACATTGCG	290
P. sizovae CBS 413.69	CGATAACTAATGTGAATTGCAGAATTCAGTGAATCATCGAGTCTTTGAACGCACATTGCG	289
	****** ***	

A. terreus QTYC38	CCCCCTGGTATTCCGGGGGGCATGCCTGTCCGAGCGTCATTGCTGCCCTCAAGCACGGCT	357
P. paxilli CBS 360.38	CCCTCTGGTATTCCGGAGGGCATGCCTGTCCGAGCGTCATTGCTGCCCTCAAGCACGGCT	351
P. argentinense CBS 130371	CCCTCTGGTATTCCGGAGGGCATGCCTGTCCGAGCGTCATTGCTGCCCTCAAGCACGGCT	348
P. pasqualense CBS 126330	CCCTCTGGTATTCCGGAGGGCATGCCTGTCCGAGCGTCATTGCTGCCCTCCAGCCCGGCT	347
P. wellingtonense DTO 76C6	CCCTCTGGTATTCCGGAGGGCATGCCTGTCCGAGCGTCATTGCTGCCCTCCAGCCCGGCT	347
P. vancouverense CBS 126323	CCCTCTGGTATTCCGGAGGGCATGCCTGTCCGAGCGTCATTGCTGCCCTCCAGCCCGGCT	347
P. ubiqetum CBS 126437	CCCTCTGGTATTCCGGAGGGCATGCCTGTCCGAGCGTCATTGCTGCCCTCCAGCCCGGCT	348
P. manginii CBS 253.31	CCCTCTGGTATTCCGGAGGGCATGCCTGTCCGAGCGTCATTGCTGCCCTCCAGCCCGGCT	348
P. pancosmium CBS 276.75	CCCTCTGGTATTCCGGAGGGCATGCCTGTCCGAGCGTCATTGCTGCCCTCCAGCCCGGCT	348
P. atrofulvum CBS 109.66	CCCTCTGGTATTCCGGAGGGCATGCCTGTCCGAGCGTCATTGCTGCCCTCCAGCCCGGCT	348
P. quebecense CBS 101623	CCCTCTGGTATTCCGGAGGGCATGCCTGTCCGAGCGTCATTGCTGCCCTCCAGCCCGGCT	348
P. cairnsence CBS 124325	CCCTCTGGTATTCCGGAGGGCATGCCTGTCCGAGCGTCATTGCTGCCCTCCAGCCCGGCT	348
P. waksmanii CBS 230.28	CCCTCTGGTATTCCGGAGGGCATGCCTGTCCGAGCGTCATTGCTGCCCTCCAGCCCGGCT	348
P. cosmopolitanum CBS 200.86	CCCTCTGGTATTCCGGAGGGCATGCCTGTCCGAGCGTCATTGCTGCCCTCCAGCCCGGCT	348
P. sumartrense CBS 281.36	CCCTCTGGTATTCCGGAGGGCATGCCTGTCCGAGCGTCATTGCTGCCCTCAAGCACGGCT	348
P. gallaicum CBS 167.81	CCCTCTGGTATTCCGGAGGGCATGCCTGTCCGAGCGTCATTGCTGCCCTCAAGCCCGGCT	346
P. copticola CBS 127355	CCCTCTGGTATTCCGGAGGGCATGCCTGTCCGAGCGTCATTGCTGCCCTCAAGCACGGCT	346
P. terrigenum CBS 127354	CCCTCTGGTATTCCGGAGGGCATGCCTGTCCGAGCGTCATTGCTGCCCTCAAGCACGGCT	346
P. hetheringtonii CBS 122392	CCCTCTGGTATTCCGGAGGGCATGCCTGTCCGAGCGTCATTGCTGCCCTCAAGCCCGGCT	313
P. citrinum NRRL 1841	CCCTCTGGTATTCCGGAGGGCATGCCTGTCCGAGCGTCATTGCTGCCCTCAAGCCCGGCT	313
P. citrinum MEF133A	CCCTCTGGTATTCCGGAGGGCATGCCTGTCCGAGCGTCATTGCTGCCCTCAAGCCCGGCT	313
P. citrinum 2.2.5.7	CCCTCTGGTATTCCGGAGGGCATGCCTGTCCGAGCGTCATTGCTGCCCTCAAGCCCGGCT	313
P. citrinum JF 11	CCCTCTGGTATTCCGGAGGGCATGCCTGTCCGAGCGTCATTGCTGCCCTCAAGCCCGGCT	313
P. citrinum S36	CCCTCTGGTATTCCGGAGGGCATGCCTGTCCGAGCGTCATTGCTGCCCTCAAGCCCGGCT	313
***P. citrinum* castor**	CCCTCTGGTATTCCGGAGGGCATGCCTGTCCGAGCGTCATTGCTGCCCTCAAGCCCGGCT	313
P. gorlenkoanum CBS 408.69	CCCTCTGGTATTCCGGAGGGCATGCCTGTCCGAGCGTCATTGCTGCCCTCAAGCACGGCT	350
P. steckii CBS 260.55	CCCTCTGGTATTCCGGAGGGCATGCCTGTCCGAGCGTCATTGCTGCCCTCAAGCACGGCT	349
P. tropicum CBS 112584	CCCTCTGGTATTCCGGAGGGCATGCCTGTCCGAGCGTCATTGCTGCCCTCAAGCACGGCT	350
P. tropicoides CBS 122410	CCCTCTGGTATTCCGGAGGGCATGCCTGTCCGAGCGTCATTGCTGCCCTCAAGCACGGCT	350
P. sizovae CBS 413.69	CCCTCTGGTATTCCGGAGGGCATGCCTGTCCGAGCGTCATTGCTGCCCTCAAGCACGGCT	349
	*** ************ ********************************* *** *****	

A. terreus QTYC38	TGTGTGTTGGGCCCCCGTCC-CCGGTTTCCCCCGGGGACGGGCCCGAAAGGCAGCGGCGG	416
P. paxilli CBS 360.38	TGTGTGTTGGGCCCTCGTCCCCC---------CGGGGACGGGCCCGAAAGGCAGCGGCGG	402
P. argentinense CBS 130371	TGTGTGTTGGGCCACCGTCCC---CCGTCCGCGGGGGACGGGCCCGAAAGGCAGCGGCGG	405
P. pasqualense CBS 126330	GGTGTGTTGGGCCCCGTCCC----CCTTCCCGGGGGGACGGGCCCGAAAGGCAGCGGCGG	403
P. wellingtonense DTO 76C6	GGTGTGTTGGGCCCCGTCCC----CCTTCCCGGGGGGACGGGCCCGAAAGGCAGCGGCGG	403
P. vancouverense CBS 126323	GGTGTGTTGGGCCCCGTCCC----CCTTCCCGGGGGGACGGGCCCGAAAGGCAGCGGCGG	403
P. ubiqetum CBS 126437	GGTGTGTTGGGCCCCGCCCC----CCTTCCCGGGGGGGCGGGCCCGAAAGGCAGCGGCGG	404
P. manginii CBS 253.31	GGTGTGTTGGGCCCCGCCCC----CCTTCCCGGGGGGGCGGGCCCGAAAGGCAGCGGCGG	404
P. pancosmium CBS 276.75	GGTGTGTTGGGCCCCGCCCC----CCTTCCCGGGGGGGCGGGCCCGAAAGGCAGCGGCGG	404
P. atrofulvum CBS 109.66	GGTGTGTTGGGCCCCGTCCC----CCTTCCCGGGGGGACGGGCCCGAAAGGCAGCGGCGG	404
P. quebecense CBS 101623	GGTGTGTTGGGCCCCGCCCC----CCTTCCCGGGGGGGCGGGCCCGAAAGGCAGCGGCGG	404
P. cairnsence CBS 124325	GGTGTGTTGGGTCCCGCCCC----CCTTCCCGGGGGGGCGGGCCCGAAAGGCAGCGGCGG	404
P. waksmanii CBS 230.28	GGTGTGTTGGGCCCCGCCCC----CCTTCCCGGGGGGGCGGGCCCGAAAGGCAGCGGCGG	404
P. cosmopolitanum CBS 200.86	GGTGTGTTGGGCCCCGCCCC----CCTTCCCGGGGGGGCGGGCCCGAAAGGCAGCGGCGG	404
P. sumartrense CBS 281.36	TGTGTGTTGGGCCCCCGTCCCCCCTCTGCCGGGGGGACGGGCCCGAAAGGCAGCGGCGG	408
P. gallaicum CBS 167.81	TGTGTGTTGGGCCCCGTCCCCCC----------GGGGACGGGCCCGAAAGGCAGCGGCGG	396
P. copticola CBS 127355	TGTGTGTTGGGCCCCCGCCCCCCC-CGCGCTGGGGGGGCGGGCCCGAAAGGCAGCGGCGG	405
P. terrigenum CBS 127354	TGTGTGTTGGGCCCCCGCCCCCC--CGCACCGGGGGGGCGGGCCCGAAAGGCAGCGGCGG	404
P. hetheringtonii CBS 122392	TGTGTGTTGGGCCCCGTCC-CCCCCGCC---GGGGGGACGGGCCCGAAAGGCAGCGGCGG	369
P. citrinum NRRL 1841	TGTGTGTTGGGCCCCGTCC-CCCCCGCC---GGGGGGACGGGCCCGAAAGGCAGCGGCGG	369
P. citrinum MEF133A	TGTGTGTTGGGCCCCGTCC-CCCCCGCC---GGGGGGACGGGCCCGAAAGGCAGCGGCGG	369
P. citrinum 2.2.5.7	TGTGTGTTGGGCCCCGTCC-CCCCCGCC---GGGGGGACGGGCCCGAAAGGCAGCGGCGG	369
P. citrinum JF 11	TGTGTGTTGGGCCCCGTCC-CCCCCGCC---GGGGGGACGGGCCCGAAAGGCAGCGGCGG	369
P. citrinum S36	TGTGTGTTGGGCCCCGTCC-CCCCCGCC---GGGGGGACGGGCCCGAAAGGCAGCGGCGG	369
***P. citrinum* castor**	TGTGTGTTGGGCCCCGTCC-CCCCCGCC---GGGGGGACGGGCCCGAAAGGCAGCGGCGG	369
P. gorlenkoanum CBS 408.69	TGTGTGTTGGGCCCCGTCC-CCCCCTCCGCG-GGGGGACGGGCCCGAAAGGCAGCGGCGG	408
P. steckii CBS 260.55	TGTGTGTTGGGCCCCGTCC-CCCCCGCTCCGGGGGGGACGGGCCCGAAAGGCAGCGGCGG	408
P. tropicum CBS 112584	TGTGTGTTGGGCCCCGTCC-CCCCCGCGCCGG-GGGGACGGGCCCGAAAGGCAGCGGCGG	408
P. tropicoides CBS 122410	TGTGTGTTGGGCCCCGTCC-CCCCCGCGCCGG-GGGGACGGGCCCGAAAGGCAGCGGCGG	408
P. sizovae CBS 413.69	TGTGTGTTGGGCCCCGTCC-CCCCCGCGCCGG-GGGGACGGGCCCGAAAGGCAGCGGCGG	407
	********** * * **** **********************	

A. terreus QTYC38	CACCGCGTCCGGTCCTCGAGCGTATGGGGCTTTGTCACCCGCTCTGTAGGCCCGGCCGGC	476
P. paxilli CBS 360.38	CACCGCGTCCGGTCCTCGAGCGTATGGGGCTTCGTCACCCGCTCGTTAGGCCCGGCGCGG	462
P. argentinense CBS 130371	CACCGCGTCCGGTCCTCGAGCGTATGGGGCTTCGTCACCCGCTCTTGCAGGCCCGGCCGG	465
P. pasqualense CBS 126330	CACCGCGTCCGGTCCTCGAGCGTATGGGGCTTTGTCACCCGCTCTTGTAGGCCCGGCCGG	463
P. wellingtonense DTO 76C6	CACCGCGTCCGGTCCTCGAGCGTATGGGGCTTTGTCACCCGCTCTTGTAGGCCCGGCCGG	463
P. vancouverense CBS 126323	CACCGCGTCCGGTCCTCGAGCGTATGGGGCTTTGTCACCCGCTCTTGTAGGCCCGGCCGG	463
P. ubiqetum CBS 126437	CACCGCGTCCGGTCCTCGAGCGTATGGGGCTTTGTCACCCGCTCTTGCAGGCCCGGCCGG	464
P. manginii CBS 253.31	CACCGCGTCCGGTCCTCGAGCGTATGGGGCTTTGTCACCCGCTCTTGCAGGCCCGGCCGG	464
P. pancosmium CBS 276.75	CACCGCGTCCGGTCCTCGAGCGTATGGGGCTTTGTCACCCGCTCTTGCAGGCCCGGCCGG	464
P. atrofulvum CBS 109.66	CACCGCGTCCGGTCCTCGAGCGTATGGGGCTTTGTCACCCGCTCTTGTAGGCCCGGCCGG	464
P. quebecense CBS 101623	CACCGCGTCCGGTCCTCGAGCGTATGGGGCTTTGTCACCCGCTCTTGTAGGCCCGGCCGG	464
P. cairnsence CBS 124325	CACCGCGTCCGGTCCTCGAGCGTATGGGGCTTTGTCACCCGCTCTTGTAGGCCCGGCCGG	464
P. waksmanii CBS 230.28	CACCGCGTCCGGTCCTCGAGCGTATGGGGCTTTGTCACCCGCTCTTGTAGGCCCGGCCGG	464
P. cosmopolitanum CBS 200.86	CACCGCGTCCGGTCCTCGAGCGTATGGGGCTTTGTCACCCGCTCTTGTAGGCCCGGCCGG	464
P. sumartrense CBS 281.36	CACCGCGTCCGGTCCTCGAGCGTATGGGGCTTCGTCACCCGCTCTTGTAGGCCCGGCCGG	468
P. gallaicum CBS 167.81	CACCGCGTCCGGTCCTCGAGCGTATGGGGCTTCGTCACCCGCTCTTGCAGGCCCGGCCGG	456
P. copticola CBS 127355	CACCGCGTCCGGTCCTCGAGCGTATGGGGCTTCGTCACCCGCTCTTGTAGGCCCGGCCGG	465
P. terrigenum CBS 127354	CACCGCGTCCGGTCCTCGAGCGTATGGGGCTTCGTCACCCGCTCTTGTAGGCCCGGCCGG	464
P. hetheringtonii CBS 122392	CACCGCGTCCGGTCCTCGAGCGTATGGGGCTTCGTCACCCGCTCTTGTAGGCCCGGCCGG	429
P. citrinum NRRL 1841	CACCGCGTCCGGTCCTCGAGCGTATGGGGCTTCGTCACCCGCTCTAGTAGGCCCGGCCGG	429
P. citrinum MEF133A	CACCGCGTCCGGTCCTCGAGCGTATGGGGCTTCGTCACCCGCTCTAGTAGGCCCGGCCGG	429
P. citrinum 2.2.5.7	CACCGCGTCCGGTCCTCGAGCGTATGGGGCTTCGTCACCCGCTCTAGTAGGCCCGGCCGG	429
P. citrinum JF 11	CACCGCGTCCGGTCCTCGAGCGTATGGGGCTTCGTCACCCGCTCTAGTAGGCCCGGCCGG	429
P. citrinum S36	CACCGCGTCCGGTCCTCGAGCGTATGGGGCTTCGTCACCCGCTCTAGTAGGCCCGGCCGG	429
***P. citrinum* castor**	CACCGCGTCCGGTCCTCGAGCGTATGGGGCTTCGTCACCCGCTCTAGTAGGCCCGGCCGG	429
P. gorlenkoanum CBS 408.69	CACCGCGTCCGGTCCTCGAGCGTATGGGGCTCTGTCACCCGCTCTTGTAGGCCCGGCCGG	468
P. steckii CBS 260.55	CACCGCGTCCGGTCCTCGAGCGTATGGGGCTTCGTCACCCGCTCTTGTAGGCCCGGCCGG	468
P. tropicum CBS 112584	CACCGCGTCCGGTCCTCGAGCGTATGGGGCTTCGTCACCCGCTCTTGTAGGCCCGGCCGG	468
P. tropicoides CBS 122410	CACCGCGTCCGGTCCTCGAGCGTATGGGGCTTCGTCACCCGCTCTTGTAGGCCCGGCCGG	468
P. sizovae CBS 413.69	CACCGCGTCCGGTCCTCGAGCGTATGGGGCTTCGTCACCCGCTCTTGTAGGCCCGGCCGG	467
	******************************* *********** * ** * *	

```
A. terreus QTYC38             GCCAG-CCGACACCCAA-----CTTTATTTCTAAGGTTGACCTCGGATCAG   521
P. paxilli CBS 360.38         CGCCAGCCGACCCCCCCTCAAT---CTTTAACCAGGTTGACCTCGGATCAG   510
P. argentinense CBS 130371    CGCCAGCCGACCCCCCTCAACCAAACTTCTC-CAGGTTGACCTCGGATCAG   515
P. pasqualense CBS 126330     CGCCAGCCGACCCCCTCAATCTATTTTTCA--GG-TTGACC-TCGGATCAG   510
P. wellingtonense DTO 76C6    CGTCAGCCGACCCCCTCAATCTATTTTTCA--GG-TTGACC-TCGGATCAG   510
P. vancouverense CBS 126323   CGCCAGCCGACCCCCTCAATCTATTTTTCA--GG-TTGACC-TCGGATCAG   510
P. ubiqetum CBS 126437        CGCCAGCCGACCCCCTCAATCTATTTTTCA--GG-TT-GACCTCGGATCAG   511
P. manginii CBS 253.31        CGCCAGCCGACCCCCTCAATCTATTTTTTC--AG-GTTGACCTCGGATCAG   512
P. pancosmium CBS 276.75      CGCCAGCCGACCCCCTCAATCTATTTTTTC--AG-GTTGACCTCGGATCAG   512
P. atrofulvum CBS 109.66      CGTCAGCCGACCCCCTCAATCTATTTTTTC--AG-GTTGACCTCGGATCAG   512
P. quebecense CBS 101623      CGCCAGCCGACCCCCTCAATCTATTTTTTC--AG-GTTGACCTCGGATCAG   512
P. cairnsence CBS 124325      CGCCAGCCGACCCCCTCAATCTATTTTTTC--AG-GTTGACCTCGGATCAG   512
P. waksmanii CBS 230.28       CGTCAGCCGACCCCCTCAATCTATTTTTTC--AG-GTTGACCTCGGATCAG   512
P. cosmopolitanum CBS 200.86  CGCCAGCCGACCCCCTCAATCTATTTTTTC--AG-GTTGACCTCGGATCAG   512
P. sumartrense CBS 281.36     CGCCAGCCGACCCCAACCCTAAATTTTTTT--CAGGTTGACCTCGGATCAG   517
P. gallaicum CBS 167.81       CGCTTGCCGACCCCCTCAATCTATTTTTCT--CAGGTTGACCTCGGATCAG   505
P. copticola CBS 127355       CGCCAGCCGACCCCCCTCAATCTATTTTTT--CAGGTTGACCTCGGATCAG   514
P. terrigenum CBS 127354      CGCCAGCCGACCCCCCTCAATCTATTTTTT--CAGGTTGACCTCGGATCAG   513
P. hetheringtonii CBS 122392  CGCCAGCCGACCCCCAACCTTTAACTATCT--CAGGTTGACCTCGGATCAG   478
P. citrinum NRRL 1841         CGCCAGCCGACCCCCAACCTTTAATTATCT--CAGGTTGACCTCGGATCAG   478
P. citrinum MEF133A           CGCCAGCCGACCCCCAACCTTTAATTATCT--CAGGTTGACCTCGGATCAG   478
P. citrinum 2.2.5.7           CGCCAGCCGACCCCCAACCTTTAATTATCT--CAGGTTGACCTCGGATCAG   478
P. citrinum JF 11             CGCCAGCCGACCCCCAACCTTTAATTATCT--CAGGTTGACCTCGGATCAG   478
P. citrinum S36               CGCCAGCCGACCCCCAACCTTTAATTATCT--CAGGTTGACCTCGGATCAG   478
P. citrinum castor            CGCCAGCCGACCCCCAACCTTTAATTATCT--CAGGTTGACCTCGGATCAG   478
P. gorlenkoanum CBS 408.69    CGCCAGCCGACCCCAAC-CTTTAATTATTTCTCAGGTTGACCTCGGATCAG   518
P. steckii CBS 260.55         CGCCAGCCGACCCCCAACCTTTTATTTTTCTCAGGTTGACCTCGGATCAG   519
P. tropicum CBS 112584        CGCCAGCCGACCCCCAACCTTTAAATT-TTCTCAGGTTGACCTCGGATCAG   518
P. tropicoides CBS 122410     CGCCAGCCGACCCCCAACCTTTAAATT-TTCTCAGGTTGACCTCGGATCAG   518
P. sizovae CBS 413.69         CGCCAGCCGACCCCCAACCTTTATATT-TTCTCAGGTTGACCTCGGATCAG   517
                                    ***** **                      *   * *********
```
