# Supplementary figures and images for: Assessment and verification of commercially available pressure cookers for laboratory sterilization
Source: PLoS One. 2018 Dec 11;13(12):e0208769. doi: 10.1371/journal.pone.0208769 (PMC6289433; doi:10.1371/journal.pone.0208769)

Before sterilization

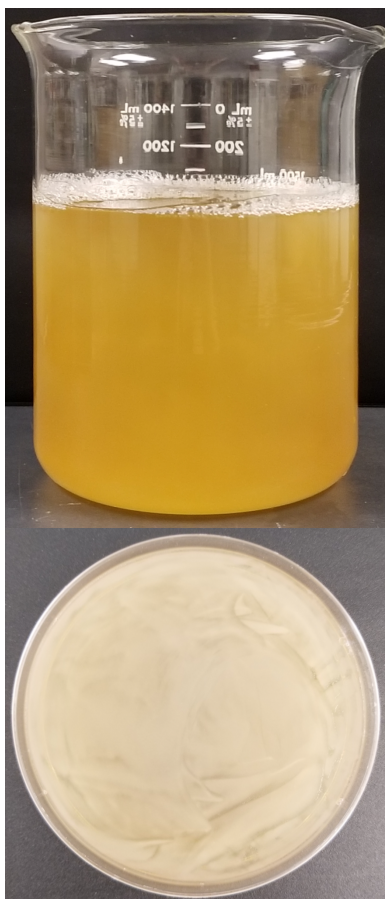

After sterilization

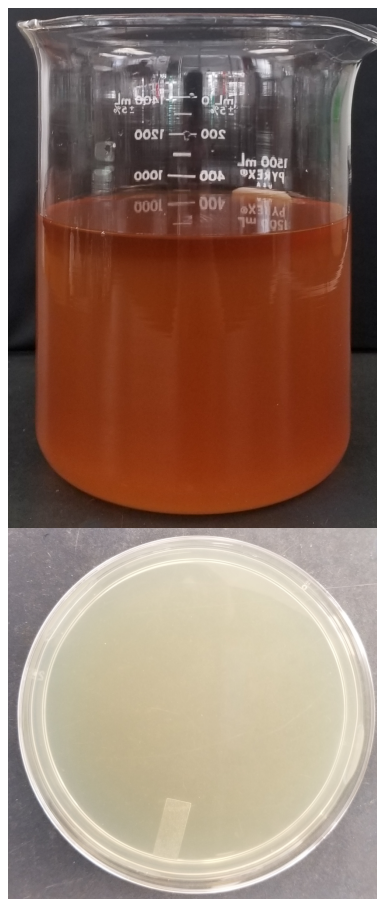

Supplement: S2 Fig — Representative culture before (left panels) and after (right panels) pressure cooker sterilization. Bacillus subtilis was grown in nutrient broth overnight with aeration and the culture is visibly turbid (upper left panel), and produced a lawn of growth when spread on a nutrient agar plate (lower left panel). After pressure cooker sterilization, the broth was a caramel color with increased sedimentation at the bottom of the beaker and a nearly transparent layer at the top (upper right panel). Complete optical transparency was not achieved following sterilization in a pressure cooker or an autoclave likely because the cytoplasmic contents of lysed cells increased the opacity of the broth. No growth was visible when the pressure cooker-sterilized culture was spread on a nutrient agar plate (bottom right panel). (PDF) [file pone.0208769.s002.pdf]
