# Supplementary material for: Assessment and verification of commercially available pressure cookers for laboratory sterilization
Source: PLoS One. 2018 Dec 11;13(12):e0208769. doi: 10.1371/journal.pone.0208769 (PMC6289433; doi:10.1371/journal.pone.0208769)
Supplement: S1 Table — Transcription of the text on the ampoules in Fig 2. (DOCX) [file pone.0208769.s003.docx]

| **Ampoule Type** | **Writing on the ampoule** |
| --- | --- |
| Negative Control | Negative Control  LOT# 16319860 |
| *Geobacillus stearothermophilus* spores | Spore Ampoule 1 mL  *G. stearothermophilus*  Steam  LOT# S1717704 |
